# Bunyaviral Cap-Snatching Endonuclease Activity and Inhibition with Baloxavir-like Inhibitors in the Context of Full-Length L Proteins

**DOI:** 10.3390/v17030420

**Published:** 2025-03-14

**Authors:** Arlo J. Loutan, Baiuyan Yang, Gabrielle Connolly, Adam Montoya, Robert J. Smiley, Arnab K. Chatterjee, Matthias Götte

**Affiliations:** 1 Department of Medical Microbiology and Immunology, Faculty of Medicine and Dentistry, University of Alberta, Edmonton, AB T6G 2E1, Canadarjsmiley@ualberta.ca (R.J.S.); 2 Calibr-Skaggs Institute for Innovation Medicines at Scripps Research, La Jolla, CA 92037, USA; byang@scripps.edu (B.Y.);

**Keywords:** bunyaviruses, cap-snatching, nuclease activity, viral RNA-dependent RNA polymerase, RdRp, baloxavir, antiviral compounds, recombinant proteins, gel-based assays

## Abstract

The *Bunyavirales* order includes a range of zoonotic viruses, which can cause severe disease in humans. The viral replication machinery is a logical target for the development of direct-acting antivirals. Inhibition of the cap-snatching endonuclease activity of related influenza viruses provides a proof of concept. Using the influenza B virus (IBV) RNA-dependent RNA polymerase complex as a benchmark, we conducted a comparative analysis of endonuclease activities of recombinant full-length bunyaviral L proteins using gel-based assays. The IBV complex demonstrates specific endonucleolytic cleavage and a clear preference for capped substrates. In contrast, severe fever with thrombocytopenia syndrome, Sin Nombre, and Hantaan virus L proteins readily cleave capped and uncapped RNAs to a broader spectrum of RNA fragments. Active site mutants further help to control for the potential of contaminating nucleases, exonuclease activity, and RNA hydrolysis. The influenza cap-snatching inhibitor baloxavir and derivatives have been used to validate this approach. In conclusion, the results of this study demonstrate the importance of assays with single nucleotide resolution and the use of full-length L proteins as a valuable experimental tool to identify selective endonuclease inhibitors.

## 1. Introduction

**Figure 1:**
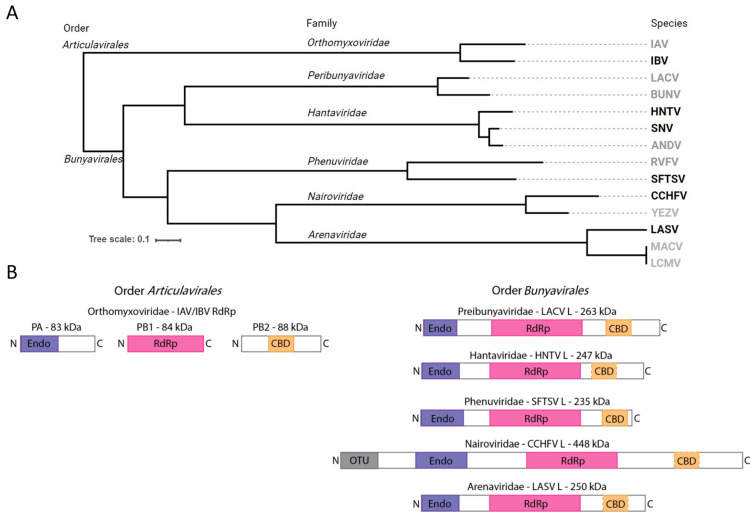
Diversity and organization of viral RdRps from selected snsRNA viruses. (**A**) Rooted phylogenetic tree based on RdRp protein sequences demonstrating the evolutionary relationships between selected human pathogenic group members. Emphasis is placed on species relevant to this study. Order, family, and species are listed beneath respective headings. Members with viral proteins employed in the present study are shown in black, those without are shown in grey. Viral species abbreviations used: Influenza A virus (IAV), Influenza B virus (IBV), LaCrosse virus (LACV), Bunyawerma virus (BUNV), Hantaan virus (HNTV), Sin Nombre virus (SNV), Andes virus (ANDV), Rift Valle fever virus (RVFV), severe fever with thrombocytopenia syndrome virus (SFTSV), Crimean–Congo hemorrhagic fever virus (CCHFV) Yezo virus (YEZV), Lassa fever virus (LASV), Machupo virus (MACV), lymphocytic choriomeningitis virus (LCMV). (**B**) Schematic diagram of viral RdRp protein complexes to illustrate relative size, heterotrimeric versus monomeric composition, and putative functional components including Endo (purple), RdRp (magenta), CBD (orange, dashed border indicates that the domain is unconfirmed), and ovarian tumor-like (OTU) domains.

Segmented negative-sense RNA (snsRNA) viruses encompass a range of human pathogens, including influenza and bunyaviruses (Figure 1A), posing a major threat to human health globally [1]. Influenza A (IAV) and B viruses (IBV) belong to the family *Orthomyxoviridae* (order *Articulavirales*) and are widely recognized for their seasonal infection cycles and pandemic potential [2,3,4]. The order *Bunyavirales* includes many arthropod- and rodent-borne zoonotic pathogens from the families *Arenaviridae*, *Nairoviridae*, *Phenuviridae*, *Hantaviridae*, and *Peribunyaviridae* with high epidemic potential [1]. Lassa fever virus (LASV, family *Arenaviridae*), Rift Valley fever virus (RVFV, family *Phenuviridae*), and Crimean–Congo hemorrhagic fever virus (CCHFV, family *Nairoviridae*) were recognized as priority pathogens by the World Health Organization (WHO) in 2017, though so far, no specific, direct-acting antivirals are available for the treatment of any bunyavirus infection.

The viral replication machinery is a logical target for antivirals [5]. SnsRNA viruses use cap-snatching to prime viral transcription, resulting in 5′ methyl-7 guanylate (m^7^G) capped viral mRNAs that are readily recognized for translation [6]. Prototypical influenza viruses carry out cap-snatching using a heterotrimeric RNA-dependent RNA polymerase (RdRp) complex [7]. The polymerase acidic (PA) protein contains an N-terminal cap-snatching endonuclease, the polymerase basic (PB)1 protein contains the RdRp catalytic components, and the PB2 protein contains a cap-binding domain (CBD; Figure 1B) [8]. Host 5′ m^7^G capped RNAs are recognized and bound by the CBD, positioning the RNA in proximity to the cap-snatching endonuclease. The RNA is cleaved by PA to a length of 10–15 nt, leaving a 3′OH [9]. The 3′ end of the cleaved host RNA is transferred to the RNA polymerase active site where it is used as a primer for the synthesis of viral mRNAs. In contrast, bunyaviral replication and transcription employ a monomeric RdRp protein encoded by the large (L) genome segment [10]. These multifunctional enzymes commonly contain an N-terminal endonuclease [11,12], central RdRp motifs [10], and a CBD in the C-terminal arm (Figure 1B) [13,14,15]. This conserved architecture resembles a sequentially linked version of influenza virus PA, PB1, and PB2 in a single protein molecule [16]. SnsRNA cap-snatching endonucleases belong to the PD-D/ExK superfamily and are expected to proceed through a two-metal-cation mechanism [12,17,18]. However, a detailed understanding of the cap-snatching process remains elusive for most bunyaviruses, limiting our ability to design potent direct-acting antivirals.

Baloxavir marboxil (BXM) is a first-in class influenza antiviral that inhibits the PA endonuclease in vitro [19] and consequently reduces disease severity [20] and decreases transmission post-exposure in humans [21]. BXM first gained U.S. Food and Drug Administration (FDA) approval in 2018 for the treatment of uncomplicated influenza virus infections [22]. The active form, baloxavir acid (BXA), confers its antiviral effect through two structural domains. The anchor domain is composed of three oxygen atoms which chelate the two divalent metal cations in the endonuclease active site [23,24,25]. The other part of BXA comprises the specificity domain, which provides primarily van der Waals interactions with residues in the binding pocket. These collective interactions allow BXA to act as a tight-binding inhibitor of the influenza virus cap-snatching endonuclease [24]. The conservation of the PD-D/ExK active site and comparable binding mode seen in structures of isolated bunyavirus endonuclease domains with related compounds suggests a promising avenue for development of bunyaviral endonuclease inhibitors [16,26,27,28]. Structural modifications to BXA, such as the addition of a 7-carboxyl adjacent to the anchor domain, appear to be important for the inhibition of bunyavirus replication [29,30]. Most studies examining cap-snatching endonuclease activity employ a truncated version of the protein containing minimal components [12,18,31]. In instances where full-length bunyaviral L proteins have demonstrated endonuclease activity, the nature of products remains elusive [14,15,32,33].

Here, we employed a comparative approach to characterize recombinant full-length bunyaviral L proteins in gel-based assays with particular focus on the endonuclease activity. The potential for contaminating nucleases, exonuclease activity, or non-enzymatic RNA hydrolysis can challenge monitoring of bona fide endonuclease activity. While IBV RdRp is tightly restricted in its specific endonuclease activity, severe fever with thrombocytopenia syndrome virus (SFTSV), Sin Nombre virus (SNV), and Hantaan virus (HNTV) L proteins show a much broader spectrum of cleavage products. CCHFV and LASV L proteins lack a clear cap-snatching endonuclease activity. Although the cap-snatching endonucleases of SFTSV and SNV L are inhibited by BXA, the potency is substantially lower. A 7-carboxyl substitution improves inhibition of SNV endonuclease relative to BXA, but not the SFTSV or IBV endonucleases. Our results highlight the importance of using gel-based monitoring of nuclease activity and its inhibition with full-length L protein across the order *Bunyavirales*.

## 2. Materials and Methods

### 2.1. Chemicals

5′ triphosphorylated oligos were sourced from ChemGenes (Wilmington, MA, USA). Baculovirus expression constructs synthesized by GenScript. All other oligos were sourced from Dharmacon (Lafayette, CO, USA). Vaccinia capping reagents were sourced from New England BioLabs (Whitby, ON, Canada). Baloxavir acid (BXA) was sourced from MedChemExpress (Monmouth Junction, NJ, USA) as a 10 mM stock in 100% DMSO. NTPs were purchased from GE Healthcare (Cranbury, NJ, USA). [α-^32^P]GTP was purchased from PerkinElmer (Boston, MA, USA). BXA derivatives were synthesized as follows with stock solutions in 100% DMSO used in the biochemical assays.

kCOT909: A suspension of (R)-7-(benzyloxy)-3,4,12,12a-tetrahydro-1H-[1,4]oxazino [3,4-c]pyrido[2,1-f][1,2,4]triazine-6,8-dione (100 mg, 305 μmol) and 7,8-difluoro-6,11-dihydrodibenzo[b,e]thiepin-11-ol (161 mg, 611 μmol) in Propylphosphonic anhydride (50% in EtOAc) (3 mL) was heated at 100 °C for 16 h. The reaction was cooled to room temperature, and directly purified by reverse phase chromatography (C18 SiO_2_, 10 to 100% MeCN in water) to give (R)-12-((R)-7,8-difluoro-6,11-dihydrodibenzo[b,e]thiepin-11-yl)-7-hydroxy-3,4,12,12a-tetrahydro-1H-[1,4]oxazino[3,4c]pyrido[2,1-f] [1,2,4]triazine-6,8-dione (3 mg, 6 μmol) as a white powder. Eluted as peak 2 of 2. Characterization data: 1H NMR (400 MHz, DMSO) δ 7.52 (d, J = 7.8 Hz, 1H), 7.35 (t, J = 7.5 Hz, 1H), 7.23 (t, J = 8.4 Hz, 3H), 7.13–7.00 (m, 2H), 5.70 (s, 1H), 5.62 (d, J = 7.6 Hz, 1H), 5.53 (d, J = 14.7 Hz, 1H), 4.43 (d, J = 13.3 Hz, 1H), 4.20 (dd, J = 10.0, 3.1 Hz, 1H), 4.09 (d, J = 14.6 Hz, 1H), 3.96 (dd, J = 10.8, 3.0 Hz, 1H), 3.71–3.61 (m, 2H), 3.42 (d, J = 11.4 Hz, 1H), 2.67 (s, 1H). *m*/*z*: 484.11.

kCOT912: Step 1: A suspension of (R)-7-(benzyloxy)-3,4,12,12a-tetrahydro-1H-[1,4]oxazino[3,4-c]pyrido[2,1-f][1,2,4]triazine-6,8-dione (100 mg, 305 μmol) and 6,11-dihydrodibenzo[b,e]thiepin-11-ol (139 mg, 611 μmol) in Propylphosphonic anhydride (50% in EtOAc) (3 mL) was heated at 100 °C for 3 h. The reaction was cooled to room temperature and directly purified by normal phase chromatography (SiO_2_, 0 to 10% MeOH in DCM) to give (12aR)-7-(benzyloxy)-12-(6,11- dihydrodibenzo[b,e]thiepin-11-yl)-3,4,12,12a-tetrahydro-1H-[1,4]oxazino[3,4-c]pyrido[2,1-f][1,2,4]triazine-6,8-dione (100 mg, 186 μmol, 60.9%). *m*/*z*: 538.18; Step 2: A solution of (12aR)-7-(benzyloxy)-12-(6,11-dihydrodibenzo[b,e]thiepin-11-yl)-3,4,12,12a-tetrahydro-1H-[1,4]oxazino[3,4-c]pyrido[2,1-f][1,2,4]triazine-6,8-dione (197 mg, 366 μmol) and TFA (0.5 mL) in DCM (3 mL) was heated at reflux for 6 h. The reaction was concentrated under reduced pressure and directly purified by reverse phase chromatography (C18 SiO_2_, 10 to 100% MeCN in water) to give (R)-12-((S)-6,11-dihydrodibenzo[b,e]thiepin-11-yl)-7-hydroxy-3,4,12,12a-tetrahydro-1H-[1,4]oxazino[3,4-c]pyrido[2,1-f][1,2,4]triazine-6,8-dione (peak 1, 4 mg, 9 μmol) and (R)-12-((R)-6,11-dihydrodibenzo[b,e]thiepin-11-yl)-7-hydroxy-3,4,12,12a-tetrahydro-1H-[1,4]oxazino[3,4-c]pyrido[2,1-f][1,2,4]triazine-6,8-dione (peak 2 (kCOT912), 2 mg, 4 μmol). Characterization Data 1H NMR (400 MHz, DMSO) δ 7.50 (d, J = 7.0 Hz, 1H), 7.40 (dd, J = 19.7, 10.1 Hz, 2H), 7.22–7.16 (m, 3H), 7.18–7.06 (m, 3H), 5.31 (d, J = 9.8 Hz, 1H), 4.51 (dd, J = 14.0, 3.3 Hz, 1H), 4.12–3.98 (m, 3H), 3.64 (dd, J = 14.1, 9.8 Hz, 1H), 3.24–3.10 (m, 1H), 3.00 (t, J = 13.2 Hz, 1H). *m*/*z*: 448.13.

mCOT923: Step 1: 5-(benzyloxy)-1-((tert-butoxycarbonyl)amino)-6-(methoxycarbonyl)-4-oxo-1,4-dihydropyridine-3-carboxylic acid(6.64 g, 15.9 mmol) and 2-(2,2-dimethoxyethoxy)ethan-1-amine (6.64 g, 44.5 mmol) were dissolved in THF (20mL) in a sealed vessel and stirred at 90 °C for 2.5 h. Water was added to the reaction mixture, and the mixture was acidified with 2 M hydrochloric acid and extracted with ethyl acetate. The organic layer was washed with brine and dried over sodium sulfate, and the solvent was distilled off under reduced pressure. The obtained crude product was crystallized with isopropyl ether to afford 5-(benzyloxy)-1-((tert-butoxycarbonyl)amino)-6-((2-(2,2-dimethoxyethoxy)ethyl)carbamoyl)-4-oxo-1,4-dihydropyridine-3-carboxylic acid as a white solid 5 (5.02 g, 15.9mmol, 59.1%). *m*/*z*: 536.22; Step 2: To a suspension of 5-(benzyloxy)-1-((tert-butoxycarbonyl)amino)-6-((2-(2,2-dimethoxyethoxy)ethyl)carbamoyl)-4-oxo-1,4-dihydropyridine-3-carboxylic acid (5.00 g, 9.34 mmol) in water (6.5 mL) and acetonitrile (75 mL), methanesulfonic acid (6 mL) was added. The reaction was heated at 60 °C for 3.5 h. The reaction was concentrated under reduced pressure and directly purified by reverse phase chromatography (C18 SiO_2_, 10 to 100% MeCN in water) to give 7-(benzyloxy)-6,8-dioxo-3,4,6,8,12,12a-hexahydro-1H-[1,4]oxazino[3,4-c]pyrido[2,1-f][1,2,4]triazine-9-carboxylicacid as a white solid (2.15 g, 62%). *m*/*z*: 372.12; Step 3: A suspension of 7-(benzyloxy)-6,8-dioxo-3,4,6,8,12,12a-hexahydro-1H-[1,4]oxazino[3,4-c]pyrido[2,1-f][1,2,4]triazine-9-carboxylic acid (50 mg, 130 μmol) and 7,8-difluoro-6,11-dihydrodibenzo[b,e]thiepin-11-ol (100 mg, 378 μmol) in Propylphosphonic anhydride (50% in EtOAc) (1.5 mL) was heated at 100 °C for 1.5 h. The reaction was cooled to room temperature and directly purified by normal phase chromatography (0 to 10% MeOH in DCM) to give 7-(benzyloxy)-12-(7,8-difluoro-6,11-dihydrodibenzo[b,e]thiepin-11-yl)-6,8-dioxo-3,4,6,8,12,12a-hexahydro-1H-[1,4]oxazino[3,4-c]pyrido[2,1-f][1,2,4]triazine-9-carboxylic acid (65 mg, 110 μmol). *m*/*z*: 618.15; Step 4: A solution of 7-(benzyloxy)-12-(7,8-difluoro-6,11-dihydrodibenzo[b,e]thiepin-11-yl)-6,8-dioxo-3,4,6,8,12,12a-hexahydro-1H-[1,4]oxazino[3,4-c]pyrido[2,1-f][1,2,4]triazine-9-carboxylic acid (58 mg, 94 μmol) and TFA (0.5 mL) in DCM (3 mL) was heated at reflux for 6 h. The reaction was concentrated under reduced pressure and directly purified by reverse phase chromatography (C18 SiO_2_, 10 to 100% MeCN in water) to give (R)-12-((S)-7,8-difluoro-6,11-dihydrodibenzo[b,e]thiepin-11-yl)-7-hydroxy-6,8-dioxo-3,4,6,8,12,12a-hexahydro-1H-[1,4]oxazino[3,4-c]pyrido[2,1-f][1,2,4]triazine-9-carboxylic acid (peak 1 (mCOT923), 3 mg, 6 μmol) and (R)-12-((R)-7,8-difluoro-6,11-dihydrodibenzo[b,e]thiepin-11-yl)-7-hydroxy-6,8-dioxo-3,4,6,8,12,12a-hexahydro-1H-[1,4]oxazino[3,4-c]pyrido[2,1-f][1,2,4]triazine-9-carboxylic acid (peak 2, 2 mg, 4 μmol). kCOT923: 1H NMR (400 MHz, DMSO) δ 14.46 (s, 1H), 12.43 (s, 1H), 8.01 (s, 1H), 7.48–7.40 (m, 2H), 7.11 (dt, J = 14.4, 7.7 Hz, 2H),6.97 (d, J = 7.7 Hz, 1H), 6.84 (t, J = 7.3 Hz, 1H), 5.88 (s, 1H), 5.47 (d, J = 14.7 Hz, 1H), 4.66 (dd, J = 9.9, 3.1 Hz, 1H), 4.46 (d, J = 13.0 Hz, 1H), 4.11 (d, J = 14.4 Hz, 1H), 4.02 (dd, J = 10.9, 3.2 Hz, 1H), 3.79 (t, J = 10.6 Hz, 1H), 3.71 (d, J = 10.4 Hz, 1H), 3.44 (t, J = 11.4Hz, 1H), 3.14 (t, J = 12.3 Hz, 1H). *m*/*z*: 528.10.

kCPF855 patent: CN112778330A—Pyridone-containing polycyclic derivative inhibitor, and preparation method and application thereof—Google Patents.

### 2.2. Protein Expression and Purification

Viral RdRp protein sequences were codon optimized for insect cells, synthesized as DNA sequences (GenScript, Piscataway, NJ, USA), and cloned into pFastBac1 (Invitrogen, Burlington, ON, Canada) plasmid. IBV RdRp was expressed as described previously wherein tobacco etch virus (TEV) protease ORF is included at the N-terminus of the open reading frame followed by linker sequences, a TEV cut site, 8x His tag, then PA (AAU94844), PB1 (AAU94857), and PB2 (AAU94870), each separated by TEV cut sites and linker sequences [9]. SFTSV L (ADX31993) is C-terminally tagged with 8XHis. SNV L (AIA08878) and HNTV L (CAA39394) are tagged N-terminally with 8X His. These plasmids were used as a starting point for baculovirus production and protein expression based on the MultiBac (Geneva Biotech, Indianapolis, IN, USA) expression system for protein expression in *Spodoptera frugiperda* insect cells (Sf9, Invitrogen, Burlington, ON, Canada) according to previously described protocols [34,35]. Catalytically inactive RdRps: IBV -Endo (PA E81Q, D109N, E120Q), IBV -Pol (PB1 D444N), SFTSV -Endo (L D112A), SFTSV -Pol (L D1126A), SNV -Endo (L E54Q, D97N, E110Q), SNV -Pol (L D1099N); HNTV -Endo (L D97A), HNTV -Pol (L D1098A, D1099A); CCHFV (AIE16126 [36]) -Endo (L E642A, E656A); LASV (AIT17397) -Endo (L E51Q, D89N, E102Q).

Proteins were purified from pelleted protein-expressing Sf9 insect cells were lysed in lysis buffer (100 mM Tris pH 8, 1 M NaCl, 5 mM TCEP, 1.0% Tween-20, 20 mM Imidazole, 10% glycerol, Roche complete protease inhibitor cocktail (MiliporeSigma Canada Ltd., Oakville, ON, Canada)) with mechanical pressure using a glass homogenizer. Lysate was separated by centrifugation at 30 k × g 4 °C for 30 min, then transferred to a His-Pur^TM^ Ni-NTA column (Thermo Fischer Scientific, Rockford, IL, USA) to be bind for 1 h. Columns were washed with 20 mM imidazole wash/elution buffer (100 mM Tris pH 8.0, 1 M NaCl, 5 mM TCEP, 0.01% Tween-20, 10% Glycerol), then eluted with 50, 100, 200, and 400 mM imidazole wash/elution buffers in series. Collected fractions were concentrated using Amicon^®^ Ultra Centrifugal Filters with 100 kDa MWCO (MilliporeSigma Canada Ltd., Oakville, ON, Canada). Protein concentration was estimated using A280. Purity was confirmed by running ~2.5 µg of purified protein on 4–15% mini-PROTEAN TGX precast gels alongside molecular weight marker (Precision Plus Protein™ Standards, Dual Color; Bio-Rad, Hercules, CA, USA) and staining with Coomassie Brilliant Blue G-250. Protein identity was confirmed by in-gel mass spectrometry (Dr. Jack Moore, Alberta Proteomic and Mass Spectrometry Facility, Edmonton, AB, Canada), with Hsp70 and Hsp90 being identified in a study by Tchesnokov et al. [37]. Purified proteins were mixed to 40% glycerol and stored at −20 °C with minimal loss of activity noted over the course of the study.

### 2.3. Biochemical Assays

RNA synthesis: Reaction components (25 mM Tris HCl pH 8.0, 0.2 mM EDTA, 0.5–2.0 µM Template (5′ AAAAAAGAUCGCGU 3′), 50–200 µM primer (5′ pACGC 3′), NTPs (ATP, UTP, CTP at 0.1–10 µM, [α-^32^P]GTP at 0.1µM), and ~200 nM enzyme), excluding the activating metal, were combined to a volume of 10 µL and incubated for 5 min at 30 °C. Reactions were started by adding 5 µL of the activating metal (final concentration 5 mM MgCl_2_, unless otherwise indicated), for a final reaction volume of 15 µL. Reactions were allowed to proceed for 30 min, then stopped using an equal volume (15 µL) of stopping buffer (formamide containing bromophenol blue and xyelene cyanone). Samples were boiled for 5 min at 95 °C and resolved by 20% polyacrylamide gel electrophoresis (PAGE) containing 8 M urea, 89 mM Tris base, 89 mM Boric acid, and 2 mM EDTA, using 1X TBE running buffer then visualized with phosphorimaging.

A previously described capped substrate depletion endonuclease assay was adapted for our purposes [9]. 5′ triphosphorylated oligos (20-nt: 5′ pppAAUCUAUAAUAGCAUUAUCC 3′; 13-nt: 5′ pppAAUCUAUAAUAGC 3′; 12-nt: 5′ pppAAUCUAUAAUAG 3′) were capped and radiolabeled using the vaccinia capping system and [α-^32^P]GTP, according to manufacturer’s specifications (New England Biolabs, Whitby, ON, Canada). The produced 5′ cap0 and radiolabeled RNAs were extracted with phenol chloroform, then purified using size-exclusion P6 columns (BioRad, Hercules, CA, USA), according to the manufacturer’s specifications for use in the assay or as markers. The 20-nt (excluding cap0) version was introduced at an estimated 100 nM to the purified enzyme of interest following 5 min of incubation at 30 °C in Tris HCl pH 8.0 (30 mM), divalent metal cation (Mg^2+^, 5 mM), and vRNA approximately equimolar to the predicted enzyme concentration (5′ AGUAGUAACAAGAGGGUAUUGUAUACCUCUGCUUCUGCU 3′ [9] for IBV (250 nM); 5′ ACACAGAGACGCCCAGAUG 3′ and 5′ AUCUGGGCGGUCUUUGUGU 3′ SFTSV (100 nM); 5′ UAGUAGUAGACUCCGAGAU 3′ and 5′ UUCUCGGAGCAUACUACUA 3′ SNV and HNTV (100 nM); 5′ UCUCAAAGAUAUAGCUAAGA 3′ and 5′ GGGGAUUGAUAUCUUUGAGA 3′ CCHFV (300 nM); 5′ CGCACCGGGGAUCCUAGGCA 3′ and 5′ UGCCUAGGAUCCUCGGUGCG 3′ LASV (1000 nM)). Reactions were allowed to proceed for the indicated amount of time, then stopped and resolved by Urea-PAGE as described for the RNA synthesis assay and visualized with phosphorimaging. Full-length enzymes were used at estimated protein concentrations as follows unless otherwise indicated: IBV—250 nM, SFTSV—20 nM, SNV and HNTV—80 nM, CCHFV—300 nM, LASV—700 nM. The uncapped substrate depletion assay used the same conditions, but a distinct substrate. 20 µM of RNA (5′ OH-AAUCUAUAAUAGCAUUAUCC 3′) was radiolabeled in forward reaction buffer using 10U T4-PNK (Thermo Fisher Scientific Inc., Rockford, IL, USA) and 2µM [γ-^32^P]ATP at 37 °C for 45 min. Resulting labeled RNA was extracted and purified in the same way as the capped RNAs to be used at an estimated 30 nM in the final reaction with otherwise identical conditions to the capped substrate depletion assay.

IC_50_ values of inhibitors were determined using the capped substrate depletion activity assay as above with the following modifications: 10 min of incubation (enzyme, chemical (10% of final reaction volume in 100% DMSO), MgCl_2_, vRNA, buffer, 50 mM NaCl, 0.01% Triton X-100) prior to addition of capped substrate. Detergent was used in the reaction to help rule out non-specific inhibition [38]. Reactions were stopped at 30 min for IBV, or 15 min for SFTSV and SNV. In all cases, gel image contrast was adjusted uniformly using ImageJ version 1.54f to ensure product visibility. Quantification of all substrate depletion products using Amersham ImageQuant™ TL analysis software version 8.2 provided total product proportion, which was normalized to the -Endo and DMSO controls within each replicate following transformation of compound concentration (x = log(x)) to determine IC_50_ values using log(inhibitor) vs. normalized response—variable slope in GraphPad Prism 10.4.1 (GraphPad Software, Boston, MA, USA).

### 2.4. Phylogenetic Analysis and Design of RdRp Functional Domain Diagram

Phylogenetic analysis (Figure 1A) conducted based on a previous report [39]. Multiple sequence alignment of Wuhan insect virus 15 RdRp (YP_009342465; to serve as an outgroup), IAV PB1 (ADC97079), IBV PB1 (AAU94857), LACV L (WHP37888), BUNV L (AIZ49763), HNTV L (ALI59821), SNV L (AUZ98443), ANDV L (WZH56198), RVFV L (XBW67510), SFTSV L (UGM45813), CCHFV L (ASW22375), YEZV L (WWT48703), LASV L (APT69666), MACV L (YP_010839405), and LCMV L (YP_010839405) was conducted using MAFFT and the L-INS-i algorithm [40]. An automated trimming process was run on the resulting alignment using TrimAl with its -automated1 option [41]. The trimmed alignment was then converted to phylip format and computed into a rooted phylogenetic tree using PhyML [42]. The Interactive Tree of Life (iTOL) web app [43] was used to render the resulting tree and remove the outgroup. Images and text were further edited using Inkscape version 1.2.2. Diagrams of snsRNA viral RdRp complex functional domains (Figure 1B) were designed using Adobe Illustrator version 28.7.5 approximately to scale based on structural evidence and functional domain predictions [6,7,11,12,13,14,15,44,45].

## 3. Results

### 3.1. Expression and Purification of Bunyaviral L Proteins

**Figure 2:**
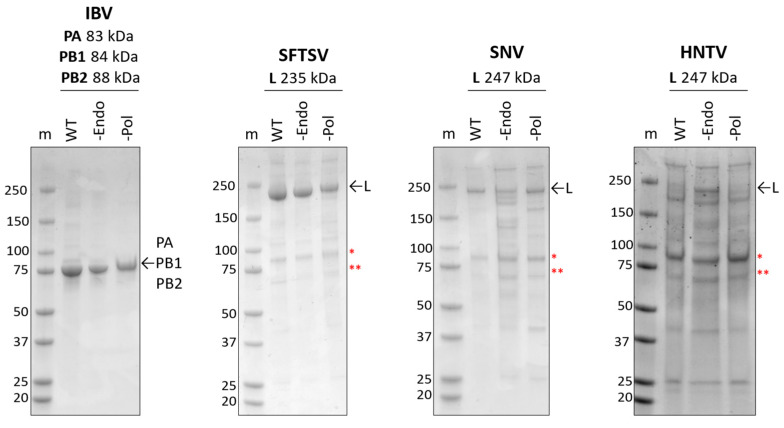
Expression and purification of bunyaviral L proteins. SDS-PAGE gel migration patterns of purified viral proteins. WT versions are unmodified relative to the reference sequence; -Endo and -Pol versions contain substitutions in active site residues to abolish the indicated activity. ~2.5 µg of protein was loaded, gels were stained with Coomassie. “m” represents the marker with marker weight in kDa listed to the left of each gel. To the right of each gel, arrows indicate purified viral protein(s), and red asterisks indicate (*) Hsp90 and (**) Hsp70, as confirmed by mass spectrometry [37].

The baculovirus system has been widely used for the expression of large viral RdRp enzymes or enzyme complexes in insect cells [15,32,36,44,45,46,47,48,49,50,51,52,53,54,55]. IBV RdRp and SFTSV (family *Phenuviridae*), SNV (family *Hantaviridae*), HNTV (family *Hantaviridae*), LASV (family *Arenaviridae*), and CCHFV (family *Nairoviridae*) L proteins were expressed in insect cells, then purified using histidine tag affinity chromatography. SDS-PAGE migration patterns of purified proteins were analyzed (Figure 2 and Figure S1A). IBV and SFTSV proteins purify to homogeneity with the expected proteins migrating to approximately 75 kDa or 230 kDa, respectively. SNV and HNTV were co-purified with more substantial host proteins, resulting in a relatively lower proportion of the L protein since protein estimates are impacted by co-purified components. L proteins were confirmed by mass spectrometry (Figure S2). In both cases, Hsp90 and Hsp70, as confirmed by mass spectrometry [37], are evident on the SDS-PAGE gel following purification. To ensure specific activities in subsequent biochemical assays, wildtype (WT), inactive endonuclease (-Endo), and inactive RNA polymerase (-Pol) versions were generated by substituting active site residues as indicated in the materials and methods. Apart from HNTV -Endo, which showed a higher proportion of the L protein compared to the WT or -Pol, all versions purified comparably. In all cases, the presence of the expected protein suggests that the bunyaviral L proteins were expressed and purified successfully.

### 3.2. Purified Proteins Demonstrate RNA Synthesis Activity

**Figure 3:**
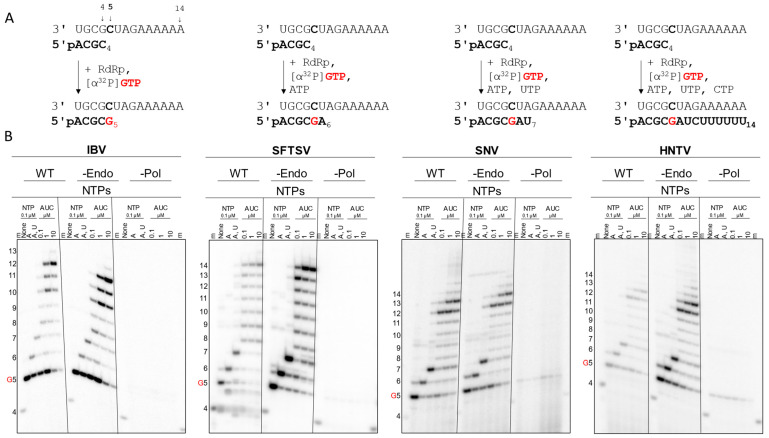
RNA synthesis activity of SFTSV, SNV, and HNTV L proteins. (**A**) Primer and template sequences and schematic of extension products based on provided NTPs. Primer is expected to base pair with template residues 1-4; the radionucleotide (α[^32^P]GTP indicated in red) is incorporated first across from the bolded template C_5_. Subsequent template nucleotides are heterogenous to allow the primer to be extended to specific products (bottom, bolded) by the addition of the indicated NTP combinations. (**B**) Gel migration patterns of RNA synthesis products by WT, -Endo, and -Pol mutant IBV, SFTSV, SNV, and HNTV viral RdRps. Purified proteins are incubated with template, primer, α[^32^P]GTP, and cold NTPs as indicated, then activated by adding MgCl_2_. Numbers to the left indicate the product size starting with the 4-nt marker “m” (radiolabeled pACGC); G5 indicates the product generated by incorporation of the radiolabeled nucleotide.

To determine whether the recombinant viral proteins were enzymatically active, an RNA synthesis assay using small model primer (4-nt) and template (14-nt) RNA sequences was employed [37]. This allowed for the control of RNA products by varying which NTPs were provided, also referred to as walking the primer along the template (Figure 3A). RNA synthesis activation of the WT proteins by various divalent metal cations was explored, but Mg^2+^ was preferred due to a decreased likelihood for artifacts or nucleotide misincorporation (Figure S3). When only the labeling α[^32^P]GTP was provided, the WT and -Endo versions of IBV, SFTSV, SNV, and HNTV synthesized the expected 5-nt product (Figure 3B). Subsequent addition of ATP or ATP and UTP resulted in the synthesis and buildup of the 6- or 7-nt products, respectively. Once ATP, UTP, and CTP were provided, the primer was extended based on the template. IBV WT and -Endo produced comparable band intensities, though the expected full-size 14-nt product was minimal and most signal accumulated at or before 12-nt. SFTSV -Endo generated greater band intensity than WT and both synthesized up to 14-nt. SFTSV WT produced some bands smaller than the predicted minimum product not seen with the -Endo, pointing to off-target cleavage of RNA by the active endonuclease. SNV WT and -Endo signal intensities were comparable, and both synthesized primarily to 12- and 13-nt when all NTPs were provided, though products at and beyond 14-nt are visible with increasing NTP concentration. HNTV -Endo produced higher band intensity than WT, but both resulted in product built up at 12- and 13-nt with some extended to 14-nt and beyond. Since there are no signs of off-target product cleavage by the endonuclease as seen with SFTSV, differences in intensity could be ascribed to differences in active site concentration across protein preparations. In all cases, the -Pol versions produced no detectable RNA products, whereas WT and -Endo specifically extend the primer according to the template and provided NTPs. CCHFV and LASV -Endo also demonstrated the expected RNA synthesis activity (Figure S1B). These results confirmed that the purified enzymes were capable of RNA synthesis.

### 3.3. Bunyaviral L Protein Cleavage Patterns Differ from the Specific IBV Endonuclease Cut

#### 3.3.1. Cleavage of Capped Substrates Lacks Strict Sequence Specificity

To confirm whether the purified proteins contained active cap-snatching endonucleases, a modified version of the substrate depletion assay described by Reich et al. [9] was used. A radiolabeled 5′ m^7^G cap0 structure—that is a 5′ to 5′ triphosphate-linked α[^32^P]m^7^GTP without 2′ OH methylation on the subsequent nucleotides—was added to a 20-nt RNA to act as the substrate. RNA derived from the extreme IBV genome ends was shown to be important for endonuclease activity; as such, we used the “mini-panhandle” RNA (terminal 18-nt from the 5′ and 3′ ends of the genome linked with 3 nt) in our assays with IBV RdRp [9]. For the bunyavirus L proteins, two RNA species representing the terminal 5′ and 3′ 19-nt from the L segment of the respective species were used to help recapitulate the probable scenario with viral RNA bound to the L protein during cap-snatching. The radiolabeled 5′ m^7^G cap0 RNA substrate was introduced to a mixture of purified viral protein, vRNA sequences, and divalent metal cation cofactor (Mg^2+^). Reactions were stopped at various time points and separated by gel electrophoresis to assess depletion products. Both band pattern and intensity must be considered to accurately gauge endonuclease products given the ubiquity of RNA nucleases and hydrolysis. Endonuclease activity is expected to result in the cleavage of the full-size substrate to smaller product(s) without buildup of intermediate products. Exonuclease activity could appear as laddering and the buildup of products at each possible size. Nuclease products end with a 3′ OH, whereas products of non-enzymatic hydrolysis contain a 3′ P, resulting in distinct gel migration patterns. IBV RdRp cleaved the substrate primarily to two products of unclear size (less than 15-mer) in a study by Reich et al. [9]. Todd et al. [24] identified one major product at 12-nt (excluding 5′ cap0) and two minor products at 14- and 11-nt.

**Figure 4:**
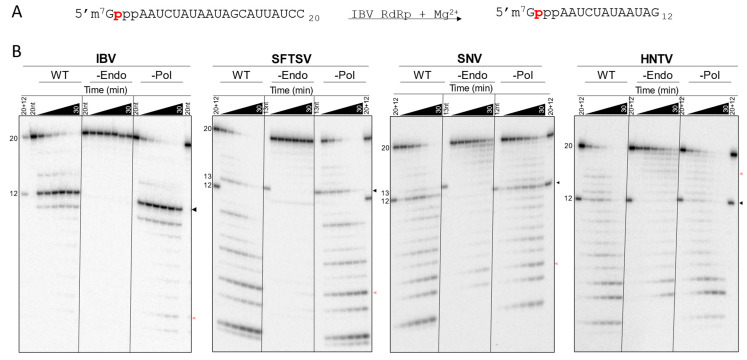
Capped substrate depletion by SFTSV, SNV, and HNTV L proteins. (**A**) Reaction scheme for IBV. The 20-nt capped and radiolabeled ([^32^P] phosphate indicated in red) substrate is cleaved primarily to a 12-nt product by IBV RdRp in the presence of Mg^2+^. (**B**) Time course of capped-substrate depletion by WT, -Endo, and -Pol viral proteins stopped at 1, 2.5, 5, 10, 20, and 30 min. Markers: capped and radiolabeled oligos of indicated length (20-nt, 12-nt, 20 + 12 (20-nt + 12-nt), or 13-nt) excluding the 5′ m^7^G cap0 residue. Sequences are as indicated in panel A: 13-nt is 12-nt + C at the 3′ end (C_13_). Arrows to the right of each gel represent specific endonuclease products (black) and non-specific nuclease products (pale red).

IBV WT and -Pol generated essentially identical substrate cleavage patterns with a major 12-nt product representing the 5′ cap and a G residue at the 3′ end (Figure 4A), and minor 11- and 14-nt (Figure 4B and Figure S4) products, as reported previously [24]. IBV -Endo lacked substrate depletion, and nearly all of the 20-nt substrate signal was retained throughout the time course. SFTSV, SNV, and HNTV WT and -Pol generated several products with relatively uniform intensity, and substrate and product depletion continued throughout the time course to yield smaller products (Figure 4B). The SFTSV -Endo L lacks detectable substrate depletion at all time points. HNTV and SNV -Endo deplete some substrate over time; however the products are distinct from WT and -Pol—mostly accumulating near the bottom of the gel—and their accumulation is delayed (Figure S4, detailed product breakdown provided in Supplementary File). The largest detectable and unique product produced by SFTSV WT and -Pol is 13-nt corresponding to a 3′ C residue, whereas for SNV and HNTV, a major product is 12-nt, as seen with IBV, suggesting some conservation in cleavage preference across snsRNA viruses. For IBV and SFTSV, the most likely explanation is that products generated by the WT and -Pol versions are the result of intrinsic endonuclease activity since active site substitution prevents their generation. SNV and HNTV WT and -Pol show distinct patterns compared to -Endo, though substrate depletion occurs in all cases. This suggests that some products result from endonuclease cleavage but that untargeted cleavage by other processes is occurring more readily with SNV and HNTV proteins. The evident ladder pattern and accumulation of small products point to exonucleolytic cleavage.

Purified isolated endonuclease domains from members of the *Arenaviridae* and *Nairoviridae* families have so far not demonstrated intrinsic endonuclease activity in vitro [12,31]. To test whether the full-length proteins may be more conducive to endonuclease activity in vitro, WT and -Endo versions of LASV and CCHFV L proteins were expressed, purified, and tested in the capped substrate depletion assay. The product banding pattern is laddered, and depletion rates are nearly identical between WT and -Endo versions for both LASV and CCHFV (Figure S1C). This suggests a lack of cap-snatching endonuclease activity in vitro. Finally, the -Endo—which lacks amino acids required for endonuclease activity—of SNV L shows some depletion of the substrate in a ladder-like pattern (Figure 4B), suggesting that this activity may be the result of co-purified nucleases, which are likely also present in the WT and -Pol. Attempts to further purify SNV WT away from potential nuclease proteins using size exclusion chromatography (SEC) were undertaken. Despite improved protein purity following SEC, the banding pattern in the substrate depletion assay remained consistent with those produced by single step purified SNV WT (Figure S5). Taken together, these results suggest that distinct products generated by SNV WT and -Pol compared to -Endo are the result of intrinsic nuclease activity. Like SFTSV, SNV has poor restriction with regard to cleavage location in vitro.

#### 3.3.2. Specific Cap-Dependent Cleavage Is Diminished with Bunyaviral Enzymes

**Figure 5:**
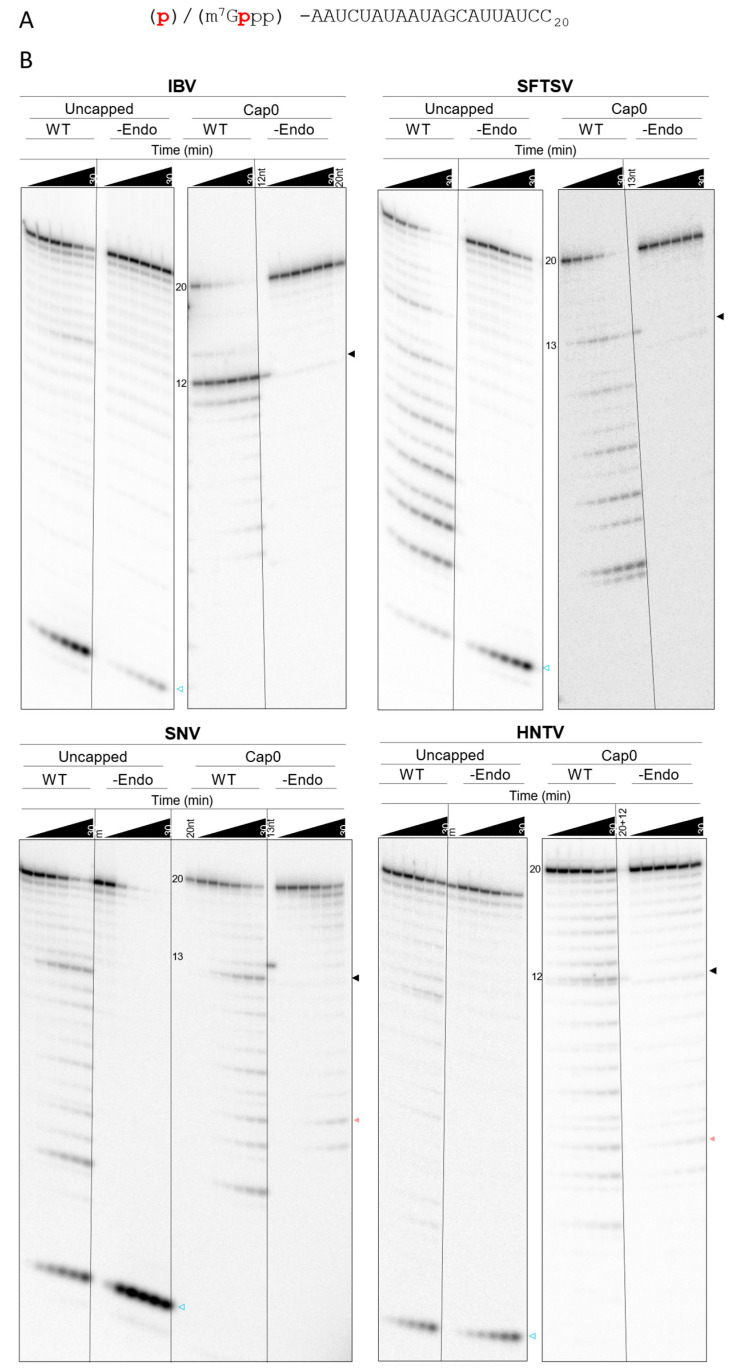
Substrate depletion of uncapped versus capped substrate by SFTSV, SNV, and HNTV L proteins. (**A**) Substrate sequence with 5′ modification and radiolabel [^32^P] indicated in red for uncapped (p) and cap0 (m^7^Gppp) substrates. (**B**) Gel migration patterns of depleted uncapped and capped substrate by IBV, SFTSV, SNV, and HNTV WT and -Endo. Reactions were performed simultaneously with recombinant proteins from each viral species using identical conditions apart from the substrate. Arrows to the right of each gel represent specific endonuclease products (black), non-specific nuclease products (pale red), and the product of 5′ to 3′ exonuclease degradation with the non-capped substrate (blue outline). Reactions stopped at 1, 2.5, 5, 10, 20, and 30 min. Markers: Cap0 markers as per Figure 4. Uncapped m = equivalent concentration of uncapped radiolabeled substrate RNA to reaction.

Prior studies mostly use isolated endonucleases with uncapped, fluorescent RNA substrate depletion assays [12]. It remains unclear whether the full-length RdRp or L protein should recognize such substrates readily given the potential presence of a CBD [13,15,56]. Indeed, IAV RdRp has previously shown decreased activity with uncapped substrates [57]. Given that a CBD for SNV and HNTV L remains unconfirmed, it was of interest to determine whether the purified viral proteins demonstrated a preference for capped RNA substrates. To this end, the depletion of a non-capped version of the RNA substrate by purified IBV, SFTSV, SNV, and HNTV proteins activated with Mg^2+^ was observed over time alongside the capped substrate (Figure 5). Analysis is like for the capped substrate, though the lack of protective 5′ m^7^G cap0 increases the likelihood of observing 5′ to 3′ exonuclease activity. Since the radioactive label is located on the 5′ end, such activity is expected to result in a product at the minimum observable size. Compared to the cap0 substrate, IBV WT only partially depleted the uncapped substrate throughout the time course, and only a faint band correlating to the major 12-nt endonuclease product is seen (Figure 5B and Figure S6). In contrast, SFTSV, SNV, and HNTV WT display comparable substrate depletion over time regardless of which substrate is provided and products generated from the uncapped substrate appear in a similar pattern to products generated from the capped substrate (Figure 5B). Some reduction in efficiency is noted between initial assessments with the capped substrate, suggesting reduction in protein activity during storage, but the pattern remains consistent. In all cases, the uncapped substrate yields a band migrating near the bottom of the gel which increases over time, representing the 5′ radiolabeled residue following 5′ to 3′ exonuclease hydrolysis. This is possibly the result of co-purified or introduced exonucleases. IBV -Endo did not deplete either substrate throughout the observed timepoints. SFTSV -Endo shows some depletion of the uncapped, but not capped, substrate. SNV -Endo shows some loss of the capped substrate in a distinct ladder pattern, whereas the uncapped substrate is largely depleted in the SNV -Endo reaction to yield the minimum product, suggesting the presence of 5′ to 3′ exonuclease activity. HNTV WT shows a higher 12-nt product signal and depletes either substrate more quickly than -Endo. Altogether, IBV endonuclease is selective for the 5′ m^7^G cap0 structure and cleavage location, whereas SFTSV, SNV, and HNTV L lack discrimination for capped RNA substrates. This suggests that bunyaviral endonucleases are not cap-dependent, or at least that factors conferring cap-dependence are absent in our conditions.

#### 3.3.3. BXA and Derivatives Can Inhibit Bunyaviral Endonuclease Activity

**Figure 6:**
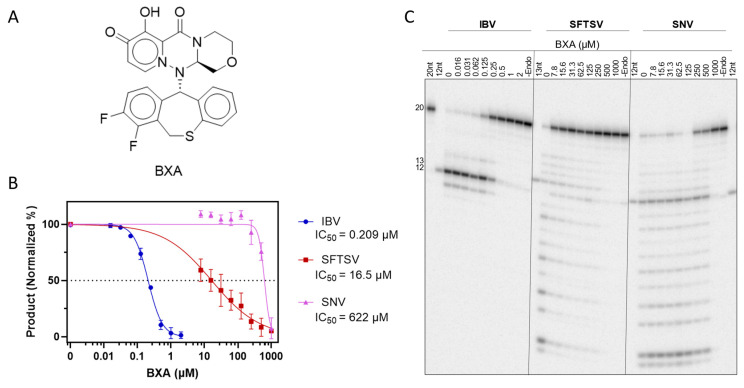
Inhibition of endonuclease activity by BXA. (**A**) Structure of BXA. (**B**) Dose–response curves of endonuclease activity vs. BXA. Calculated mean IC_50_ values (*n* = 3) displayed beneath the indicated viral protein in the legend. All products were quantified as a proportion of total band signal per lane, then normalized to the 0 (DMSO) and -Endo controls. Error bars represent standard deviation. (**C**) Representative gel migration patterns from capped substrate depletion by IBV, SFTSV, and SNV WT against BXA at indicated concentrations. Markers as per Figure 4.

The promise of cap-snatching endonuclease inhibitors against bunyaviruses led us to compare the ability of BXA (Figure 6A) to inhibit the activity of full-length L proteins in the capped substrate depletion assay (Figure 6B,C). Increasing concentrations of BXA were expected to reduce the generation of endonuclease products on the gel, allowing for the quantification and determination of half-maximal inhibitory concentration (IC_50_). HNTV was excluded due to the relatively higher prevalence of contaminating activity and difficulties in protein production. As expected, endonuclease activity of IBV was inhibited by BXA with an IC_50_ of 209 nM. SFTSV endonuclease activity was sharply reduced at micromolar BXA concentrations, but full inhibition was not seen until higher concentrations, yielding an IC_50_ of 16.5 µM. Finally, inhibition of SNV endonuclease was not evident until ~250 µM and was only inhibited at these high concentrations, yielding an IC_50_ of 622 µM. High IC_50_ values against nuclease activity compared to IBV suggest decreased affinity of BXA for bunyaviral active sites. Despite reduced potency against bunyaviral endonucleases, BXA up to 1 mM did not inhibit RNA synthesis by IBV, SFTSV, or SNV (Figure S7), suggesting that the endonuclease activity is inhibited selectively.

**Figure 7:**
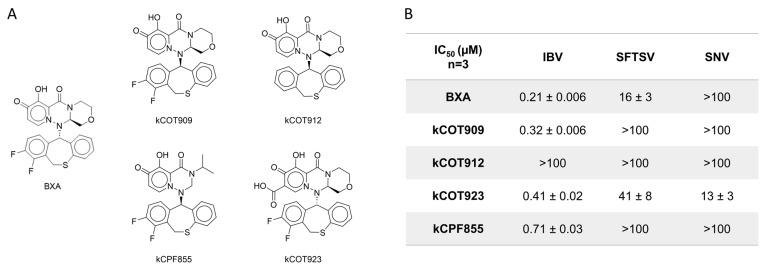
Inhibition of endonuclease activity by BXA-like compounds. (**A**) Structures of BXA and derivatives tested. kCOT923 is a racemic mixture. (**B**) Table summary of resulting IC_50_ values ± standard error calculated from 3 replicate dose–response assays performed in the capped substrate depletion assay with BXA for comparison. Values > 100 represent less than 50% inhibition by 100 µM of compound during screening for which more accurate dose–responses were not explored due to low potency.

Previous reports have highlighted a 7-carboxyl substitution as a feature that improved inhibition of bunyavirus replication [29,30]. Select BXA derivatives (Figure 7A) were synthesized and tested against IBV, SFTSV, and SNV endonuclease activity via the capped substrate depletion assay to generate IC_50_ values (Figure 7B and Figure S8A,B). Except for kCOT912, which lacks the 7,8-difluoro, all derivatives resulted in IC_50_ values sub-micromolar range against the IBV endonuclease, comparable to BXA. In contrast, inhibition of SFTSV endonuclease is abolished (IC_50_ > 100 µM) by changes in the stereochemistry of the specificity group in kCOT909 compared to BXA and not improved by the presence of the 7-carboxyl substitution in kCOT923 (16 vs. 41 µM). kCOT923 is a racemic mixture, which may result in differences to the observed IC_50_; therefore, the source of the increase in IC_50_ cannot be confidently attributed to the 7-carboxyl substitution but instead may be the result of stereochemistry. On the other hand, SNV endonuclease is not efficiently inhibited (IC_50_ >100 µM) by BXA or any derivative except for kCOT923, clearly showing the importance of the 7-carboxyl modification. As with BXA, RNA synthesis by IBV, SFTSV, and SNV is not affected by these compounds (Figure S8C,D). These results suggest that these BXA analogs act through the same metal-chelating mechanism BXA and that the 7-carboxyl substitution is important but not sufficient to improve inhibition of bunyaviral endonucleases relative to BXA in all cases.

## 4. Discussion

Given the lack of effective direct-acting antiviral therapeutics to treat infection with viruses that belong to the order *Bunyavirales*, we studied the multifunctional L protein that represents a logical target. We conducted a comprehensive comparative analysis of several full-length L proteins and used the trimeric IBV complex as a benchmark. The various enzymes represent prototypic species of important families of viruses implicated in human disease. We focused on the cap-snatching endonuclease activity and its inhibition with derivatives of BXA. While the sequence of events involved in cap-snatching and the inhibition of key activities have been described in great detail for IBV, our mechanistic understanding of the nuclease activity associated with bunyavirus enzymes is limited [6].

In this study, we included L proteins of the families *Hantaviridae* (HNTV, SNV), *Phenuviridae* (SFTSV), *Arenaviridae* (LASV), and *Nairoviridae* (CCHFV). All enzymes demonstrate polymerase activity using short primer and template RNA substrates that mimic the elongation stage in transcription or replication. Mutant enzymes (-Pol) with amino acid substitutions in the polymerase active site are essentially inactive. For the IBV complex, the WT and the -Pol mutant showed the same specific endonucleolytic cut, yielding a 12-nt, 5′-capped fragment with a 3′-end G residue. The cut is highly specific, representing sequence preference and the distance between the CBD and the endonuclease active site [9,57]. Varying the RNA sequence can change endonucleolytic cleavage location and efficiency, but the specificity of influenza virus endonuclease for cleavage between a G and C residue was previously shown to be consistent across different RNAs [57]. Two minor products representing 11- and 14-nt point to a limited degree of flexibility with the provided RNA substrate. Essentially no cuts are seen with the -Endo mutant. Cleavage patterns observed with the bunyaviral enzymes are more complex. For SFTSV, SNV, and HNTV, we observe multiple cuts with WT and the -Pol mutant. Among these cuts are a 13-nt fragment (SFTSV) and a 12-nt fragment (SNV, HNTV) that may correspond to specific endonuclease activity compatible with cap-snatching. The 12-nt fragment generated by SNV and HNTV is consistent with the high proportion of G residues at the -1 position in HNTV mRNAs during infection [58], suggesting a potential sequence preference conserved in hantavirus endonucleases. The additional fragments are likely the result of more random endo- or exonucleolytic cuts. With few exceptions, these are not seen with -Endo mutants. Contaminating nuclease activities are unlikely the source for these reactions. In addition, cap-dependent cleavage, as demonstrated for IBV, is not observed with SFTSV, SNV, and HNTV. Hantavirus endonucleases become self-limiting in overexpression systems [59]. In some reports, successful expression of full-length hantaviral L proteins has relied on mutation to the endonuclease active site [14,44]. Keown et al. [33] recently succeeded in expressing HNTV L WT, which depleted an RNA substrate in the presence of divalent metal cations unlike their -Endo control, though products were not investigated. We successfully expressed unadulterated, full-length SNV and HNTV L. While SNV was stable regardless of endonuclease activity status, the HNTV -Endo version was more easily expressed in our case, suggesting differences within the family and off-target effects or host-restriction mechanisms of the endonuclease. In contrast, LASV and CCHFV L lack clear endonuclease activity in vitro, consistent with previous reports of isolated endonucleases and full-length L proteins [12,31,47]. Williams et al. [32] recently published the structure and a corresponding biochemical assay for cap-snatching states of SFTSV L; however, the proportion of transcribed product in the assay is low, suggesting inefficiency, perhaps due to the poorly restricted nature of RNA cleavage by the endonuclease. Despite recent advances, it remains unclear how bunyaviruses use cap-snatching and which factors may be required for the process to occur efficiently.

The lack of biochemical evidence for endonuclease activity with LASV and CCHFV enzymes is an obstacle in current drug discovery and development efforts. However, the gel-based assay used in this study enables inhibition studies with SFTSV and SNV. BXA is substantially less potent against SFTSV and SNV endonuclease activity compared to IBV. Toba et al. [29] have recently shown that the addition of a 7-carboxyl group adjacent to the metal binding domain can improve antiviral effects in vitro against bunyaviruses including LASV, lymphocytic choriomeningitis virus, Junin virus, La Crosse virus, and SFTSV. We demonstrate that inhibition of the SNV-associated endonuclease activity is indeed more pronounced by a 7-carboxyl-containing compound than BXA, while inhibition of SFTSV is not. This highlights the importance of additional structural features to accurately target bunyaviral endonucleases. Collectively, these data point to challenges in the development of pan-bunyavirus inhibitors targeting the endonuclease activity. However, gel-based assays, as employed in this study, can help to discover and validate selective inhibitors that target species-specific enzymes.

## Data Availability

The original contributions presented in this study are included in the article/Supplementary Material. Further inquiries can be directed to the corresponding author.

## Supplementary Materials

The following supporting information can be downloaded at https://www.mdpi.com/article/10.3390/v17030420/s1: Figure S1: Purification and biochemical activity of CCHFV and LASV L proteins; Figure S2: Mass Spectrometry analysis of SFTSV, SNV, and HNTV L proteins; Figure S3: Effect of divalent metal cations on RNA synthesis; Figure S4: Breakdown of products produced in the capped substrate depletion assay; Figure S5: Size-exclusion chromatography purification and resulting capped substrate depletion by SNV L WT; Figure S6: Breakdown of products produced in the uncapped substrate depletion assay; Figure S7: RNA synthesis by IBV, SFTSV, and SNV WT in the presence of BXA; Figure S8: Endonuclease dose–responses and RNA synthesis by IBV, SFTSV, and SNV WT in the presence of BXA derivatives.
