# Supplementary material for: Bunyaviral Cap-Snatching Endonuclease Activity and Inhibition with Baloxavir-like Inhibitors in the Context of Full-Length L Proteins
Source: Viruses. 2025 Mar 14;17(3):420. doi: 10.3390/v17030420 (PMC11946187; doi:10.3390/v17030420)
Supplement: Supplementary file 1 [file viruses-17-00420-s001.zip › viruses-3488913-SI.pdf]

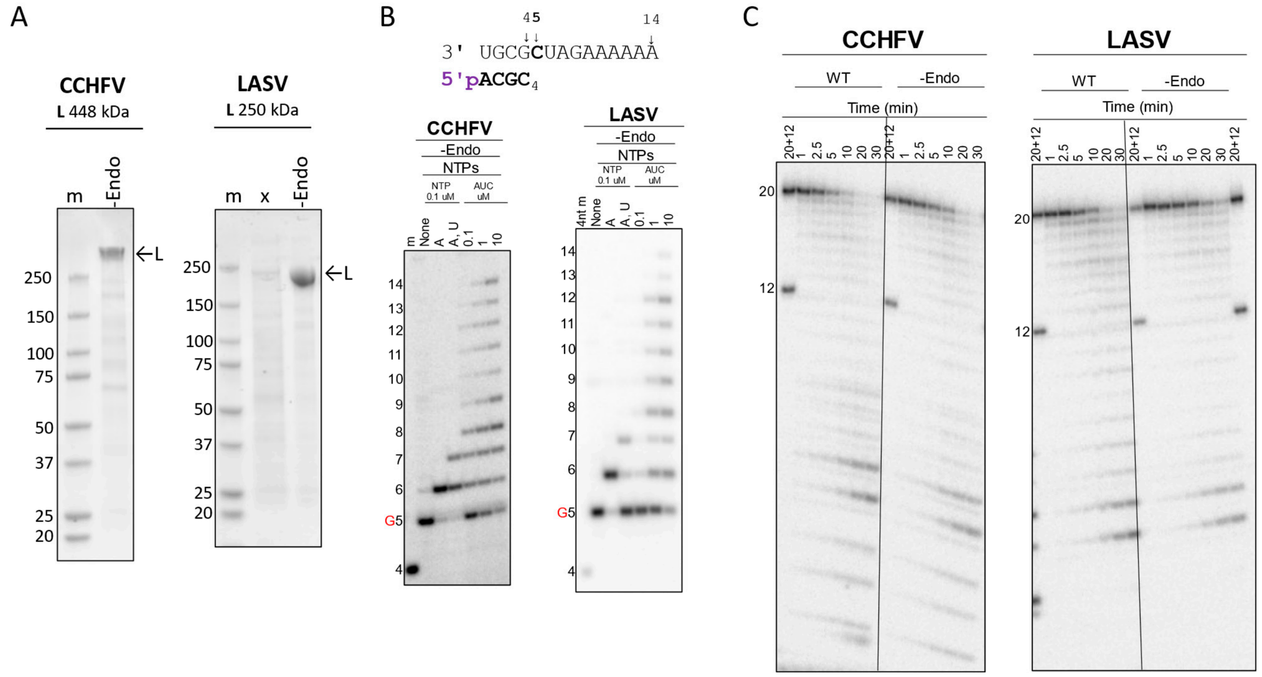

Figure S1: Purification and biochemical activity of CCHFV and LASV L proteins. (**A**) SDS-PAGE gel migration patterns of CCHFV and LASV L -Endo with L protein band indicated. (**B**) Gel migration patterns of RNA synthesis products by CCHFV and LASV L -Endo. Purified proteins are incubated with template, primer, [α-^{32}P]GTP, and cold NTPs as indicated, then activated by adding $MgCl_2$. (**C**) Capped substrate depletion assay gel migration patterns by WT and -Endo CCHFV and LASV. 20-nt capped and radiolabeled substrate is introduced to the purified protein in the presence of $MgCl_2$ and the reaction is stopped at indicated time points to assess product formation.

```
>ADX31993 SFTSV L
MNLEVLCGRINVENGLSLGEPGLYDQIYDRPGLPDLDVTVDATGVTVDIGAVPDSASQLGSSINAGLITIQLSEAYKINHDFTFSGLSKTTDRRLSEVFPITHDGSDGMTPD
VIHTRLDGTIVVVEFSTTRSHNIGGLEAAYRTKIEKYRDPISRRVDIMENPRVFFGVIVVSSGGVLSNMPLTQDEAEELMYRFCIANEIYTKARSMDADIELQKSEEELEAI
SRALSFFSLFEPNIERVEGTFPNSEIEMLEQFLSTPADVDFITKTLKAKEVEAYADLCDSHYLKPEKTIQERLEINRCEAIDKTQDLLAGLHARSNKQTSLNRGTVKLPPWL
PKPSSESIDIKTDSGFGSLMDHGAYGELWAKCLLDVSLGNVEGVVSDPAKELDIAISDDPEKDTPKEAKITYRRFKPALSSSARQEFSLQGVEGKKWKRMAANQKKEKESHE
TLSPFLDVEDIGDFLTFNNLLADSRYGDESVQRAVSILLEKASAMQDTELTHALNDSFKRNLSSNVVQWSLWVSCLAQELASALKQHCRAGEFIIKKLKFWPIYVIIKPTKS
SSHIFFSLGIRKADVTRRLTGRVFSDTIDAGEWELTEFKSLKTCKLTNLVNLPCTMLNSIAFWREKLGVAPWLVRKPCSELREQVGLTFLVSLEDKSKTEEIITLTRYTQME
GFVSPPMLPKPQKMLGKLDGPLRTKLQVYLLRKHLDCMVRIASQPFNLIPREGRVEWGGTFHAISGRSTNLENMVNSWYIGYYKNKEESTELNALGEMYKKIVEMEEDKPSS
PEFLGWEDTDSPKKHEFSRSFLRAACSSLEREIAQRHGRQWKQSLEERVLKEIGTKNILDLASMKATSNFSKDWELYSEVQTKEYHRSKLLEKMATLIEKGVMWYIDAVGQA
WKAVLDDGCMRICLFKKNQHGGLREIYVMDANARLVQFGVETMARCVCELSPHETVANPRLKNSIIENHGLKSARSLGPGSININSSNDAKKWNQGHYTTKLALVLCWFMPA
KFHRFIWAAISMFRRKKMMVDLRFLAHLSTKSESRSSDPFREAMTDAFHGNREVSWMDKGRTYIKTETGMMQGILHFTSSLLHSCVQSFYKSYFVSKLKEGYMGESISGVVD
VIEGSDDSAIMISIRPKSDMDEVRSRFFVANLLHSVKFLNPLFGIYSSEKSTVNTVYCVEYNSEFHFHRHLVRPTLRWIAASHQISETEALASRQEDYSNLLTQCLEGGASF
SLTYLIQCAQLLHHYMLLGLCLHPLFGTFMGMLISDPDPALGFFLMDNPAFAGGAGFRFNLWRACKTTDLGRKYAYYFNEIQGKTKGDEDYRALDATSGGTLSHSVMVYWGD
RKKYQALLNRMGLPEDWVEQIDENPGVLYRRAANKKELLLKLAEKVHSPGVTSSLSKGHVVPRVVAAGVYLLSRHCFRFSSSIHGRGSAQKASLIKLLMMSSVSAMKHGGSL
NPNQERMLFPQAQEYDRVCTLLEEVEHLTGKFVVRERNIVRSRIDLFQEPVDLRCKAEDLVSEVWFGLKRTKLGPRLLKEEWDKLRASFAWLSTDPSETLRDGPFLSHVQFR
NFIAHVDAKSRSVRLLGAPVKKSGGVTTISQVVRMNFFPGFSLEAEKSLDNQERLESISILKHVLFMVLNGPYTEEYKLEMIIEAFSTLVIPQPSEVIRKSRTMTLCLLSNY
LSSKGGSILDQIERAQSGTLGGFSKPQKTFIRPGGGVGYKGKGVWTGVMEDTHVQILIDGDGTSNWLEEIRLSSDARLYDVIESIRRLCDDLGINNRVASAYRGHCMVRLSG
FKIKPASRTDGCPVRIMERGFRIRELQNPDEVKMRVRGDILNLSVTIQEGRVMNILSYRPRDTDISESAAAYLWSNRDLFSFGKKEPSCSWICLKTLDNWAWSHASVLLAND
RKTQGIDNRAMGNIFRDCLEGSLRKQGLMRSKLTEMVEKNVVPLTTQELVDILEEDIDFSDVIAVELSEGSLDIESIFDGAPILWSAEVEEFGEGVVAVSYSSKYYHLTLMD
QAAITMCAIMGKEGCRGLLTEKRCMAAIREQVRPFLIFLQIPEDSISWVSDQFCDSRGLDEESTIMWG

>AIA08878 SNV L
MEKYREIHQRIKEIPPGGASALECLDLLDRLYAVRHDVVDQMIKHDWSDNEDMERPIGQVLLMAGVPNDVIQGMEKKVIPTSPSGQILKSFFRMTPDNYKITGALIEFIEVT
VTADVAKGIREKKLKYESGLQFVESLLSQEHKKGNINQAYKITFDVVAVKTDGSNISTQWPSRRNDGVVQHMRLVQADINYVREHLIKPDERASLEAMFNLKFHVGGPKLRY
FNIPDYKPQSLCQPEITNLIQYCKHWLTEDHDFVFKEVTGNNVMNSFENNEDVYMSRYRESRKPRNFLLIQGSIQGPYLPSTISSDQCDTRIGCLEVLKVHPETPVQAIAVD
MAYKYMELNRDEIINYYNPRVHFQATQSVKEPGTFKLGLSQLNPMSKSILDQVGKHKSEKGLFGEPLESINISSQIQQNECSRIIESILSNLEINVGEVTMSLANPRKTTGV
DELLGKFYENELSKYLISILRKTAAWHIGHLIRDITESLIAHAGLKRSKYWSIHAYDHGGVILFILPSKSLEVVGSYIRYFTVFKDGIGLIDEENLDSKVDIDGVQWCFSKV
MSIDLNRLLALNIAFEKALLATATWFQYYTEDQGHFPLQHALRSVFSFHFLLCVSQKMKICAIFDNLRYLIPAVTSLYSGYELLIEKFFERPFKSALEVYLYNIIKALLISL
AQNNKVRFYSKVRLLGLTVDHSTVGASGVYPSLMSRVVYKHYRSLISEATTCFFLFEKGLHGNLNEEAKIHLETVEWARKFEAKERKYGDILMREGYTIDAIRVGDVQVEQQ
LFCQEVVELSAEELNKYLQAKSQVLSSNIMNKHWDKPYFSQTRNISLKGMSGALQEDGHLAASVTLIEAIRFLNRSQTNPNVIDMYEQTKQHKAQARIVRKYQRTEADRGFF
ITTLPTRVRLEIIEDYYDAIARVVPEEYISYGGDKKILNIQTALEKALRWASGSSEVITSTGNVIKFKRRLMYVSADATKWSPGDNSAKFKRFTQALYDGLSDEKLKCCVVD
ALRHVYETEFFMSRKLHRYIDSMDEHSEAVQDFLDFFKGGVSATVKGNWLQGNLNKCSSLFGAAVSLLFRRIWAELFPELECFFEFAHHSDDALFIYGYLEPEDDGTDWFLY
VSQQIQAGNYHWHAVNQEMWKSMFNLHEHLLLMGSIKVSPKKTTVSPTNAEFLSTFFEGCAVSIPFIKILLGSLSDLPGLGFFDDLAAAQSRCVKAMDLGASPQLAQLAVVI
CTSKVERLYGTADGMVNSPVAFLKVTKAHVPIPLGGDGSMSIMELATAGIGMADKNILKQAFYSYKHTRRDGDRYVLGLFKFLMSLSEDVFQHDRLGEFSFVGKVQWKVFTP
KNEFEFFDQFSQSYLKSWTNQHPVYDYIIPRGRDNLLVYLVRKLNDPSIVTAMTMQSPLQLRFRMQAKQHMKVCKLEGEWVTFREVLAAADSFATKYNPTEKDLDLFNTLVS
CTFSKEYAWKDFLNEVRCEVVPTKHVHRSKIARTFTVREKDQAIQNPITAVIGYKYASTVDEISDVLDSSFFPDSLSADLQVMKEGVYRELGLDIGLPEVLKRIAPLLYKAG
RSRVVIVEGNVEGTAESICSYWLRSMSLVKTIKVRPKKEVLRAVSLYSTKENIGLQDDVAATRLCIEVWRWCKANDQNVNDWLNALYFEKQTLMDWVERFRRKGVVPIDPEI
QCIALLLYDVLGYKSVLQMQANRRAYSGKQYDAYCVQTYNEETRLYEGDLRVTFNFGLDCARLEIFWDKKEYILETSITQRHVLKLMMEEVTQELLRCGMRFKTEQVSHTKS
LVLFKTESGFEWGKPNVPCIVFKHCALRTGLRTKQAINKEFMINVQADGFRAIAQMDMESPRFLLAHAYHTLRDVRYQAVQAVGNVWFQTTQHKLFINPIISSGLLENFMKG
LPAAIPPAAYSLIMNKAKISVDLFMFNELLALVNPKNVLNLDGIEETSEGYSTVTSISSRQWSEEVSLMADDDIDDEEEFTIALDDIDFEQINLDEDIQHFLQDESAYTGDL
TIQTEEVEVKRIRGVTRVLEPVKLIKSWVSKGLAIDKVYNPIGIVLMARYMSKNYDFSKIPLALLNPYDLTEFESVVKGWGETVNDRFLEVDNDAQRLVREKNILPEDILPD
SLFSFRHVDVLLKRLFPRDPVSSFY

>CAA39394 HNTV L
MDKYREIHNKLKEFSPGTLTAVECIDYLDRLYAVRHDIVDQMIKHDWSDNKDSEEAIGKVLLFAGVPSNIITALEKKIIPNHPTGKSLKAFFKMTPDNYKISGTTIEFVEVT
VTADVDKGIREKKLKYEAGLTYIEQELHKFFLKGEIPQPYKITFNVVAVRTDGSNITTQWPSRRNDGVVQYMRLVQAEISYVREHLIKTEERAALEAMFNLKFNISTHKSQP
YYIPDYKGMEPIGANIEDLVDYSKDWLSRARNFSFFEVKGTAVFECFNSNEANHCQRYPMSRKPRNFLLIQCSLITSYKPATTLSDQIDSRRACSYILNLIPDTPASYLIHD
MAYRYINLTREDMINYYAPRIQFKQTQNVREPGTFKLTSSMLRAESKAMLDLLNNHKSGEKHGAQIESLNIASHIVQSESVSLITKILSDLELNITEPSTQEYSTTKHTYVD
TVLDKFFQNETQKYLIDVLKKTTAWHIGHLIRDITESLIAHSGLKRSKYWSLHSYNNGNVILFILPSKSLEVAGSFIRFITVFRIGPGLVDKDNLDTILIDGDSQWGVSKVM
SIDLNRLLALNIAFEKALIATATWFQYYTEDQGQFPLQYAIRSVFANHFLLAICQKMKLCAIFDNLRYLIPAVTSLYSGFPSLIEKLFERPFKSSLEVYIYYNIKSLLVALA
QNNKARFYSKVKLLGLTVDQSTVGASGVYPSFMSRIVYKHYRSLISEVTTCFFLFEKGLHGNMNEEAKIHLETVEWALKFREKEEKYGESLVENGYMMWELRANAELAEQQL
YCQDAIELAAIELNKVLATKSSVVANSILSKNWEEPYFSQTRNISLKGMSGQVQEDGHLSSSVTIIEAIRYLSNSRHNPSLLKLYEETREQKAMARIVRKYQRTEADRGFFI
TTLPTRCRLEIIEDYYDAIAKNISEEYISYGGEKKILAIQGALEKALRWASGESFIELSNHKFIRMKRKLMYVSADATKWSPGDNSAKFRRFTSMLHNGLPNNKLKNCVIDA
LKQVYKTDFFMSRKLRNYIDSMESLDPHIKQFLDFFPDGHHGEVKGNWLQGNLNKCSSLFGVAMSLLFKQVWTNLFPELDCFFEFAHHSDDALFIYGYLEPVDDGTDWFLFV
SQQIQAGHLHWFSVNTEMWKSMFNLHEHILLLGSIKISPKKTTVSPTNAEFLSTFFEGCAVSIPFVKILLGSLSDLPGLGYFDDLAAAQSRCVKALDLGASPQVAQLAVALC
TSKVERLYGTAPGMVNHPAAYLQVKHTDTPIPLGGNGAMSIMELATAGIGMSDKNLLKRALLGYSHKRQKSMLYILGLFKFLMKLSDETFQHERLGQFSFIGKVQWKIFTPK
SEFEFADMYTSKFLELWSSQHVTYDYIIPKGRDNLLIYLVRKLNDPSIVTAMTMQSPLQLRFRMQAKQHMKVCRLDGEWVTFREVLAAANSFAENYSATSQDMDLFQTLTSC
TFSKEYAWKDFLNGIHCDVIPTKQVQRAKVARTFTVREKDQIIQNSIPAVIGYKFAVTVEEMSDVLDTAKFPDSLSVDLKTMKDGVYRELGLDISLPDVMKRIAPMLYKSSK
SRVVIVQGNVEGTAEAICRYWLKSMSLVKTIRVKPHKEVLQAVSIFNRKEDIGQQKDLAALKLCIEVWRWCKANSAPYRDWFQALWFEDKTFSEWLDRFCRVGVPPIDPEIQ
CAALMIADIKGDYSVLQLQANRRAYSGKQYDAYCVQTYNEVTKLYEGDLRVTFNFGLDCARLEIFWDKKAYILETSITQKHVLKIMMDEVSKELIKCGMRFNTEQVQGVRHM
VLFKTESGFEWGKPNIPCIVYKNCVLRTSLRTTQAINHKFMITIKDDGLRAIAQHDEDSPRFLLAHAFHTIRDIRYQAVDAVSNVWFIHKGVKLYLNPIISSGLLENFMKNL
PAAIPPAAYSLIMNRAKISVDLFMFNDLLKLINPRNTLDLSGLETTGDEFSTVSSMSSRLWSEEMSLVDDDEELDDEFTIDLQDVDFENIDIEADIEHFLQDESSYTGDLLI
STEETESKKMRGIVKILEPVRLIKSWVSRGLSIEKVYSPVNIILMSRYISKTFNLSTKQVSLLDPYDLTELESIVRGWGECVIDQFESLDREAQNMVVNKGICPEDVIPDSL
FSFRHTMVLLRRLFPQDSISSFY
```

Figure S2. Mass Spectrometry analysis of SFTSV, SNV, and HNTV L proteins. Sequences detected by mass spectrometry are highlighted in yellow.

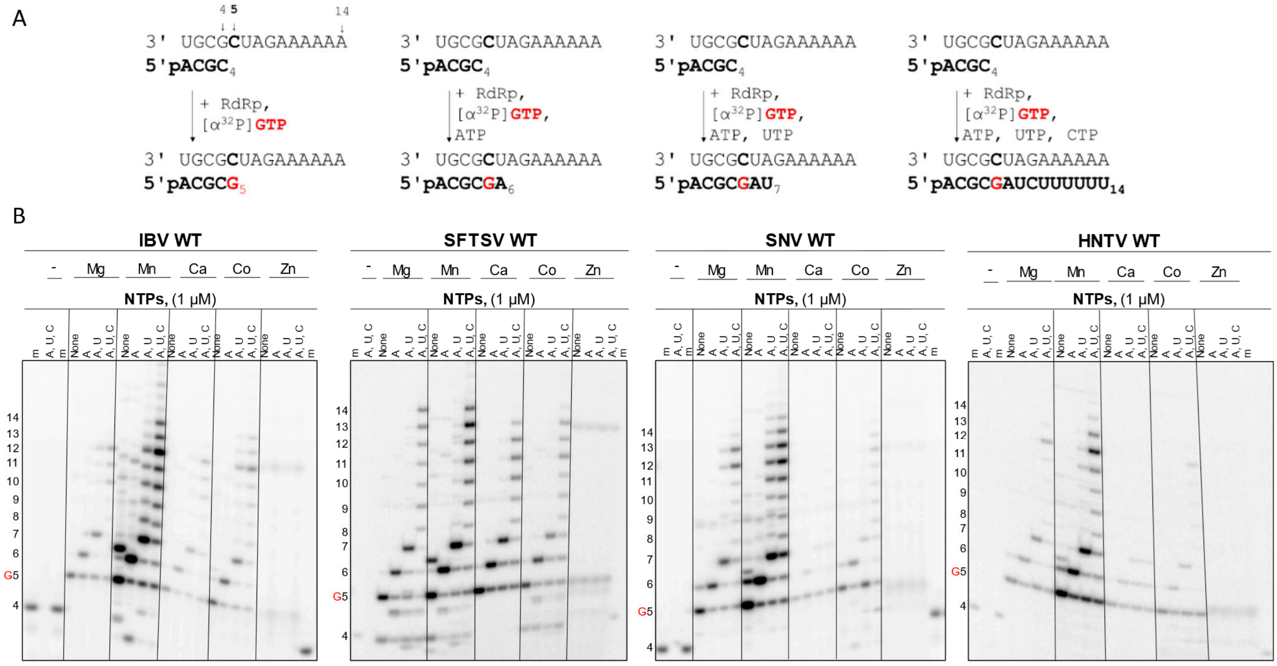

Figure S3: Effect of divalent metal cations on RNA synthesis. (A) Template and primer sequences with reaction scheme based on provided nucleotide(s). (B) Gel-migration patterns of IBV, SFTSV, SNV, and HNTV WT with indicated divalent metal cation (provided at 2.5 mM) and NTPs as indicated. Numbers to the left of each image indicate the migration location of predicted products starting with the 4-nt marker "m" (radiolabeled pACGC), G5 indicates the radiolabeled nucleotide.

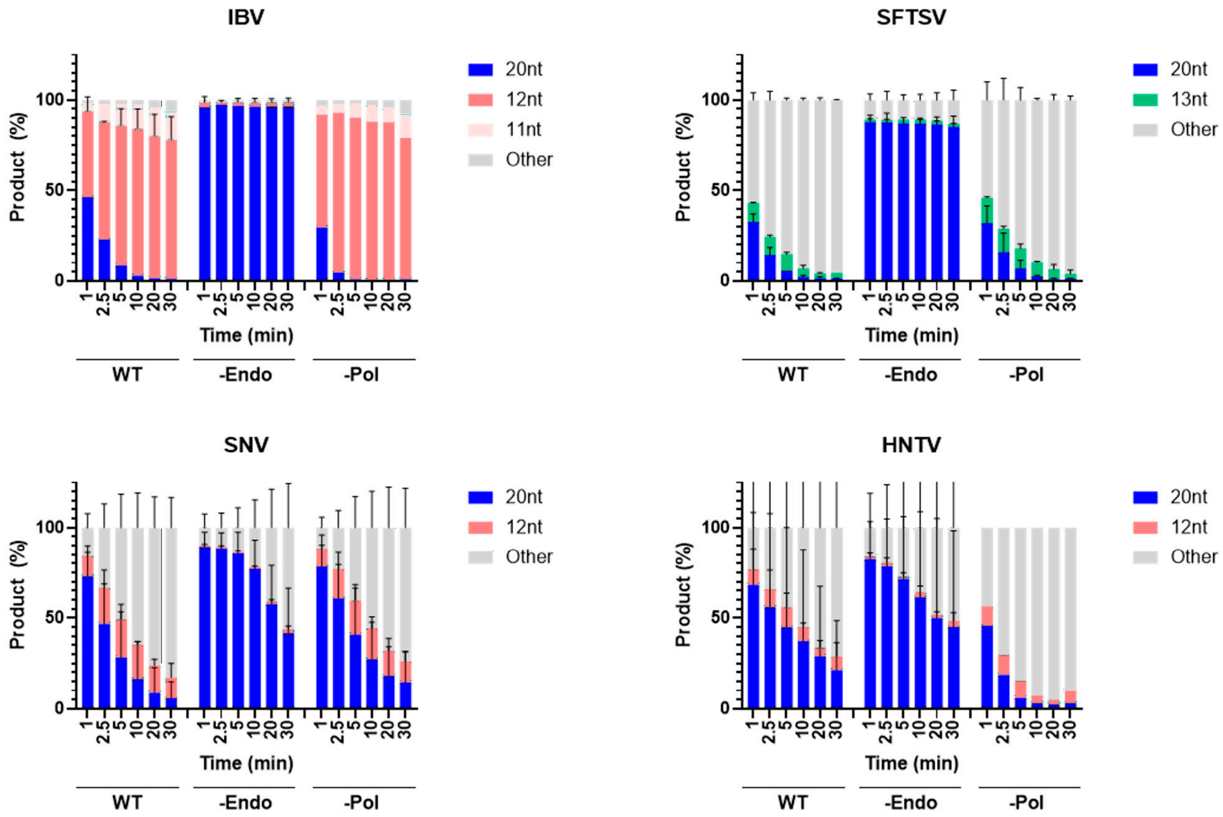

Figure S4: Breakdown of products produced in the capped substrate depletion assay. Signal intensities of bands produced in the capped substrate depletion assay were quantified to calculate percentage of indicated products out of total lane signal. The assay was repeated twice for IBV (except for -Pol), SFTSV, and HNTV (except for -Pol), or three times for SNV with error bars representing the standard deviation for each product. The 20-nt substrate is represented in blue. The 12-nt product is shown in a darker red colour with the minor 11-nt product generated by IBV in pale red. 13-nt product produced by SFTSV is shown in green. All other products are represented with grey. Detailed breakdown provided in supplementary file.

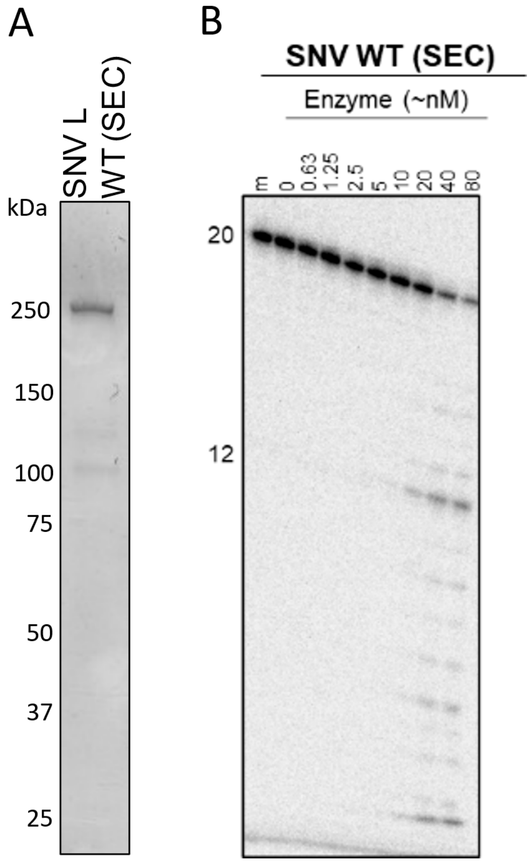

Figure S5: Size-exclusion chromatography (SEC) purification and resulting capped substrate depletion pattern of SNV L WT. (A) SDS-PAGE migration of selected SNV L WT SEC fraction. (**B**) Capped substrate depletion patterns by increasing concentration of SEC purified SNV L WT.

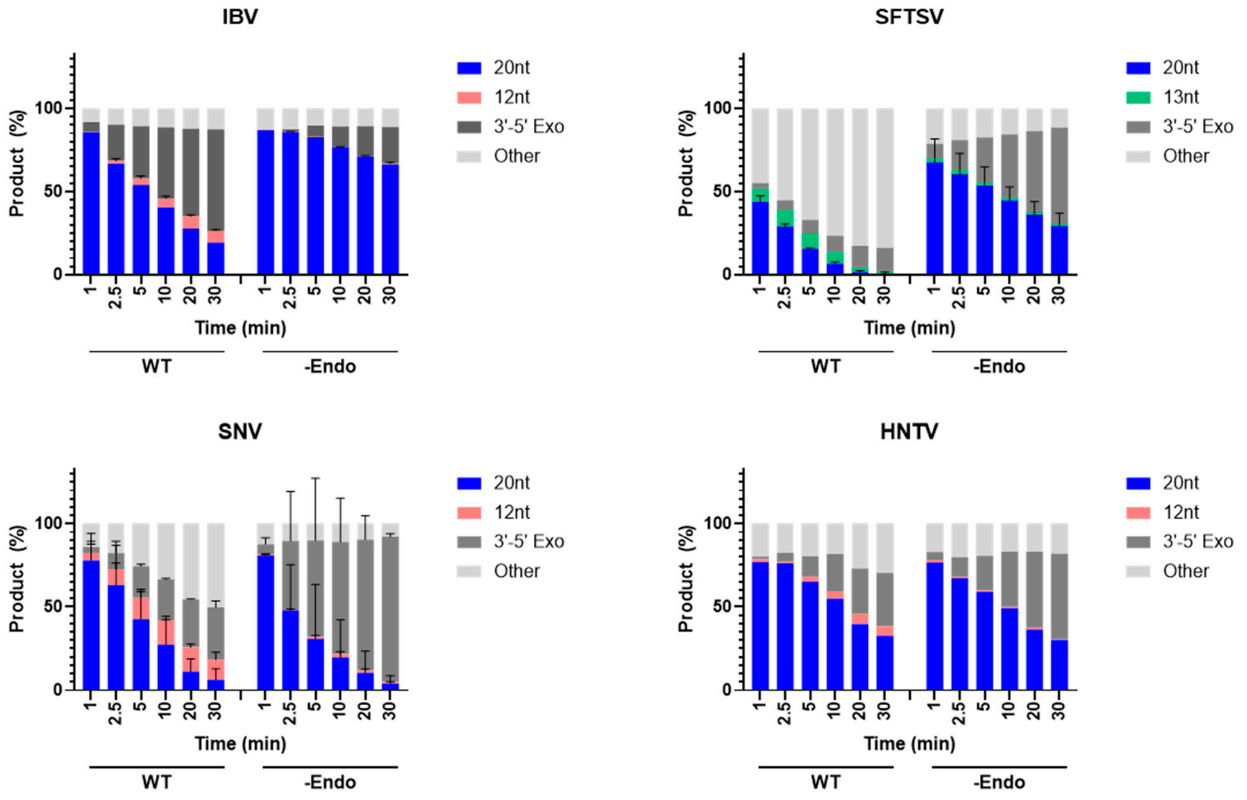

Figure S6: Breakdown of products produced in the uncapped substrate depletion assay. Signal intensities of bands produced in the capped substrate depletion assay were quantified to calculate percentage of indicated products out of total lane signal. The assay was repeated twice for IBV, SFTSV, and SNV with error bars representing the standard deviation of each band. HNTV was restricted to one replicate due to limited quantities of purified protein. The 20-nt substrate is shown in blue, major endonuclease 12-nt product in red or 13-nt product in green, and the band resulting from 3’ to 5’ exonuclease cleavage in dark grey, all other products are represented by the light grey fraction. Detailed breakdown provided in supplementary file.

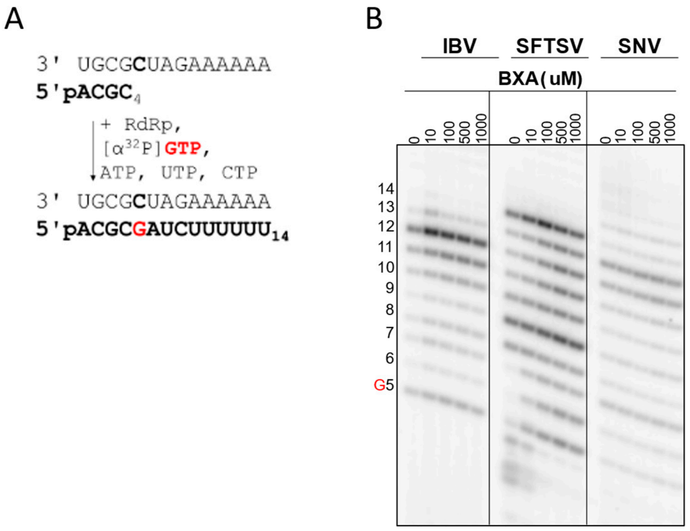

Figure S7: RNA synthesis by IBV, SFTSV, and SNV WT in the presence of BXA. (A) RNA template and primer sequences and reaction scheme. All NTPs (α[^{32}P]GTP, ATP, UTP, and CTP) provided along with 0.01% Triton X-100 to prevent non-specific inhibition. (B) Gel migration pattern resulting from RNA synthesis with increasing concentrations of BXA. No significant loss of overall signal intensity across concentrations indicates a lack of inhibition of RNA synthesis.

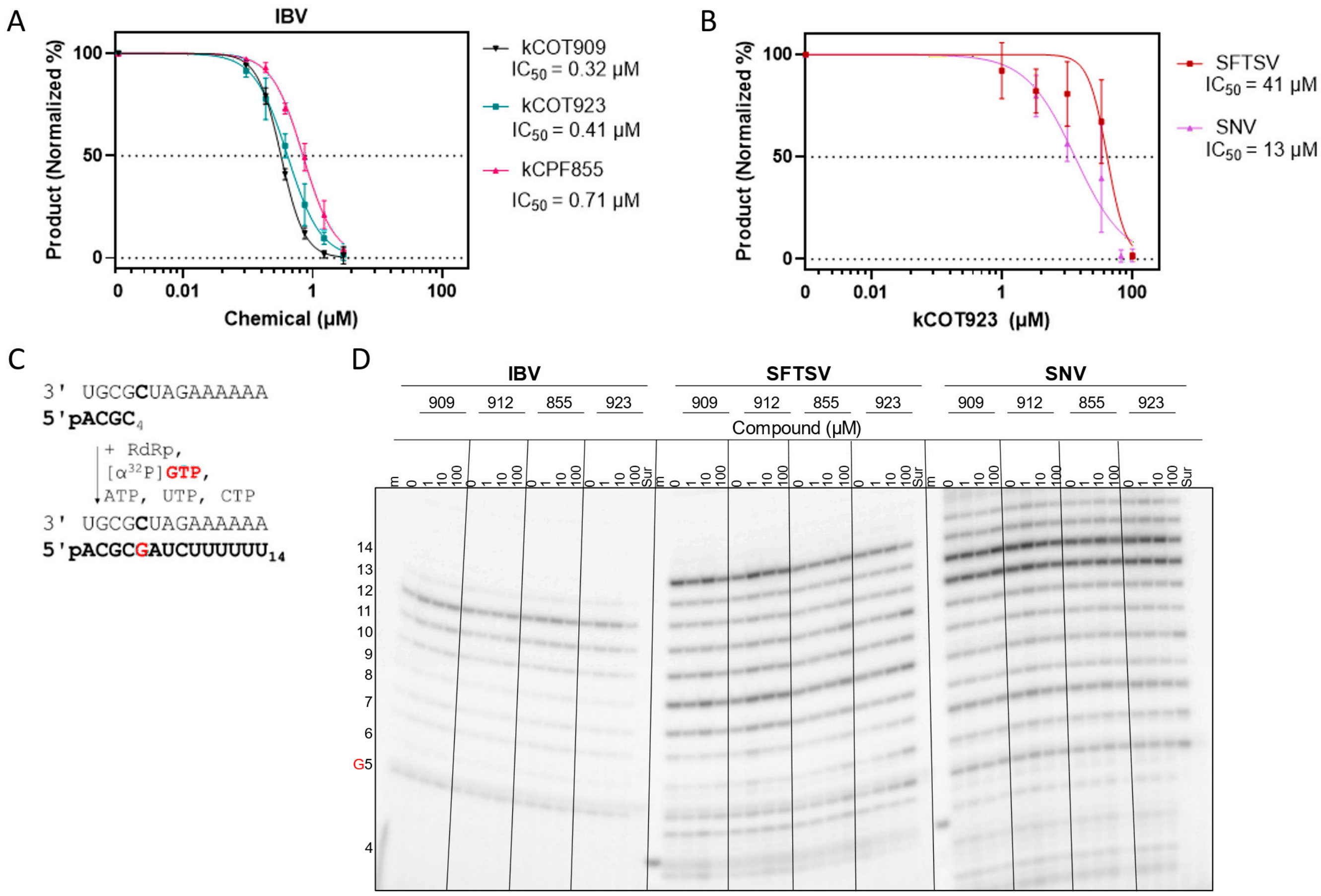

Figure S8: Endonuclease dose-responses and RNA synthesis by IBV, SFTSV, and SNV WT in the presence of BXA derivatives. (A) Dose-response curves of kCOT909, kCOT923, and kCPF855 versus IBV endonuclease activity. (B) Dose-response curves of kCOT923 versus SFTSV and SNV endonuclease activity. Error bars represent standard deviation. (C) RNA template and primer sequences and reaction scheme. All NTPs (α[^{32}P]GTP, ATP, UTP, and CTP) provided along with 0.01% Triton X-100 to prevent non-specific inhibition. (D) Gel migration patterns from RNA synthesis challenged with increasing concentrations of compounds (909 – kCOT909; 912 – kCOT912; 855 – kCPF855; or 923 – kCOT923). Sur – 100 uM suramin control. m – 4-nt (pACGC) marker. No loss of overall signal intensity with increasing compound concentrations indicates a lack of inhibition of RNA synthesis.
